# Suppression of Transforming Growth Factor‐β Signaling Delays Cellular Senescence and Preserves the Function of Endothelial Cells Derived from Human Pluripotent Stem Cells

**DOI:** 10.5966/sctm.2016-0089

**Published:** 2016-09-20

**Authors:** Hao Bai, Yongxing Gao, Dixie L. Hoyle, Tao Cheng, Zack Z. Wang

**Affiliations:** ^1^ Division of Hematology, Johns Hopkins University School of Medicine, Baltimore, Maryland, USA; ^2^ State Key Laboratory of Experimental Hematology, Institute of Hematology and Blood Disease Hospital, Chinese Academy of Medical Sciences, Tianjin, People's Republic of China; ^3^ Tianjin Key Laboratory of Blood Cell Therapy and Technology, Tianjin, People's Republic of China

**Keywords:** Endothelial cells, Human pluripotent stem cells, Senescence, Transforming growth factor‐β

## Abstract

Transplantation of vascular cells derived from human pluripotent stem cells (hPSCs) offers an attractive noninvasive method for repairing the ischemic tissues and for preventing the progression of vascular diseases. Here, we found that in a serum‐free condition, the proliferation rate of hPSC‐derived endothelial cells is quickly decreased, accompanied with an increased cellular senescence, resulting in impaired gene expression of endothelial nitric oxide synthase (eNOS) and impaired vessel forming capability in vitro and in vivo. To overcome the limited expansion of hPSC‐derived endothelial cells, we screened small molecules for specific signaling pathways and found that inhibition of transforming growth factor‐β (TGF‐β) signaling significantly retarded cellular senescence and increased a proliferative index of hPSC‐derived endothelial cells. Inhibition of TGF‐β signaling extended the life span of hPSC‐derived endothelial and improved endothelial functions, including vascular network formation on Matrigel, acetylated low‐density lipoprotein uptake, and eNOS expression. Exogenous transforming growth factor‐β1 increased the gene expression of cyclin‐dependent kinase inhibitors, p15^Ink4b^, p16^Ink4a^, and p21^CIP1^, in endothelial cells. Conversely, inhibition of TGF‐β reduced the gene expression of p15^Ink4b^, p16^Ink4a^, and p21^CIP1^. Our findings demonstrate that the senescence of newly generated endothelial cells from hPSCs is mediated by TGF‐β signaling, and manipulation of TGF‐β signaling offers a potential target to prevent vascular aging. Stem Cells Translational Medicine *2017;6:589–600*

**Significance Statement**

Endothelial cells derived from human pluripotent stem cells (hPSCs) could provide a valuable cell source for tissue engineering and treatment of cardiovascular diseases, such as revascularization of ischemic tissue. To increase the life span of hPSC‐derived endothelial cells, this study investigated expansion of hPSC‐derived endothelial cells in a serum‐free defined condition and their function for treatment of regional ischemia in an animal model. This study provides not only a reliable and unlimited source of endothelial cells for future clinical applications but also insights into molecular mechanisms of vascular aging.

## Introduction

The inability to revascularize damaged tissues occurs frequently in aging patients and those with diabetes, obesity, or hypertension, and is associated with reduced number and functionality of bone marrow‐derived circulating endothelial progenitor cells (EPCs) that play an important role in the maintenance of endothelial integrity and hemostasis 1, 2, 3, 4, 5. Revascularization by transplantation of exogenous vascular cells offers a potential noninvasive method for repair of ischemic tissues 6, 7, 8, 9. Because there is limited availability of vascular cells from human primary tissues, it is a challenge to obtain sufficient vascular cells for clinical applications for vascular regeneration, such as peripheral arterial disease. Human pluripotent stem cells (hPSCs), including human embryonic stem cells (hESCs) and induced pluripotent stem cells (iPSCs), propagate indefinitely and give rise to every cell type in the body, thereby provide attractive sources of vascular cells for vascular regeneration in ischemic tissues. Particularly, patient‐specific iPSCs are a promising source of autologous endothelial cells (ECs) for cell therapy of vascular diseases without the risk of immune rejection. We previously demonstrated that hESC‐derived endothelial cells (hESC‐ECs) are capable of self‐assembling into functional blood vessels that spontaneously integrate into the circulatory system in a mouse model 10. The vascular network formation from hESC‐ECs is regulated by SDF‐1/CXCR4 signaling 11.

A study of human induced pluripotent stem cells (hiPSCs) showed that endothelial cells and hematopoietic cells derived from hiPSCs have limited expansion potential and early senescence 12. Cellular senescence, a major cause of organismal aging, is a permanent withdrawal from the cell cycle in response to diverse stress conditions and is associated with alteration in gene expression *o*f cell cycle regulators 13, 14, 15. The cell cycle arrests induced by p53 and p16^INK4A^‐ retinoblastoma (Rb) pathways are involved in the senescence of many cell types 16. Cellular senescence not only causes somatic cells to be “mortal” in vitro but also occurs in vivo to contribute to organismal aging and other age‐related pathologies 13, 14, 15, 16. Characterization of cellular senescence of human pluripotent stem cell‐derived endothelial cells (hPSC‐ECs) provides a model system of vascular aging.

James et al. demonstrated that transforming growth factor‐β (TGF‐β) signaling plays an important role in regulating expansion and maintenance of hESC‐ECs 17. TGF‐β signaling is a multifunctional signaling pathway that regulates cell proliferation and differentiation, tissue remodeling, and repair, and it often has opposite effects dependent on the context. For example, TGF‐β is a potent growth inhibitor of the epidermis, whereas in the dermis, TGF‐β acts as a positive growth factor via induction of the synthesis of extracellular matrix proteins. TGF‐β expression is correlated with aging of several tissues and diseases, including kidney and renal disease 18, reproductive system failure 19, 20, osteoporosis 21, 22, Alzheimer's 23, adverse fibrosis of the heart 24, and other cardiovascular diseases 25. TGF‐β is a member of a superfamily that contains approximately 40 potential growth factors that are classified into 2 groups. The first group, including TGF‐β (1, 2, and 3), activins, and nodal, activates Smad2/3 transcription factors via ALK4, ALK5, and ALK7 receptors. The second group, including the bone morphogenetic proteins (BMPs) and growth differentiation factors (GDFs), binds to the ALK1, ALK2, ALK3, or ALK6 receptor and then activates downstream Smad1/5/8 26. We previously demonstrated that BMP and TGF‐β have distinct effects on hESC differentiation into vascular progenitor cells 27, 28.

Here, we report that endothelial cells derived from hemato‐endothelial progenitors (HEPs) from hPSCs have limited expansion potential in a serum‐free condition. Prolonged culture of hPSC‐ECs results in an increased senescence and impaired endothelial function. Transforming growth factor‐β1 (TGF‐β1) is expressed in hPSC‐ECs and secreted into the culture medium. Inhibition of TGF‐β signaling pathway by an inhibitor, SB431542, reduces senescent cells and preserves endothelial function in vitro and in vivo. The expansion capability of hPSC‐ECs and endothelial function significantly increases in the presence of SB431542. TGF‐β‐induced senescence is associated with downregulation of human telomerase reverse transcriptase (hTERT), longer G_1_ phase, and upregulation of p15^Ink4b^, p16^Ink4a^, and p21^CIP1^.

## Materials and Methods

### Maintenance of hPSCs

The H1 and H9 hESCs were obtained from the WiCell Research Institute (Madison, WI, http://www.wicell.org) and used between passage 40 and passage 60. The hiPSCs established from dermal fibroblast cells (HDFa‐YK26) were kind gifts from Dr. Ren‐He Xu at the University of Connecticut Health Center 29. BC1 hiPSCs, established from CD34‐positive bone marrow cells, were obtained from Dr. Linzhao Cheng at Johns Hopkins University 30. The hPSCs were cultured on vitronectin‐coated plates in E8 medium (Thermo Fisher Scientific Life Sciences, Waltham, MA, https://www.thermofisher.com) and growth medium was changed every day. The hPSCs were subcultured every 4 to 5 days with a treatment of 0.5 mM EDTA for passaging.

### Differentiation of Embryoid Bodies

The hPSC differentiation was induced, as previously described 27, 28. To generate hanging‐drop embryoid bodies (EBs), 1,000 cells per drop in 25 μl was cultured on Petri dishes to form EBs in the presence of 10 μM ROCK inhibitor (Y27632), 2 ng/ml TGF‐β1, and 0.05% polyvinyl alcohol in a serum‐free differentiation medium (SFM) containing IMDM/F12 (1:1), 0.5% bovine serum albumin (BSA; Sigma‐Aldrich, St. Louis, MO, https://www.sigmaaldrich.com), 1% ITS‐X (Thermo Fisher), 1% chemically defined lipid (Thermo Fisher), 2 mM GlutaMAX (Thermo Fisher), 50 μg/ml l‐ascorbic acid (Sigma‐Aldrich), and 450 μM 1‐thioglycerol (Sigma‐Aldrich). After 2 days of hanging‐drop EB formation, EBs were collected and transferred to ultra‐low attachment dishes in SFM. The differentiation medium was changed every 2 days with a fresh supplement of growth factors, BMP4, FGF2, and VEGF, as well as TGF‐β inhibitor, SB431542, in a time‐frame as previously described 28.

### Culture of hPSC‐Endothelial Cells

After 8 to 10 days of EB differentiation, EBs were treated with TrypLE (Thermo Fisher) at room temperature for 10 minutes to obtain a single‐cell suspension. The HEPs, CD34^+^CD31^+^CD144^+^ cells, were isolated by fluorescence‐activated cell sorting (FACS) with FACSAria II (BD Biosciences, Franklin Lakes, NJ, http://www.bdbiosciences.com). In some experiments, the CD34^+^ cells were positively selected by using MultiSort immunomagnetic separation system (Miltenyi Biotec, San Diego, CA, http://www.miltenyibiotec.com) following the manufacturer's instruction. The isolated CD34^+^CD31^+^CD144^+^ cells were cultured on a collagen‐I coated 6‐well plate (1 × 10^5^ cells per well) in serum‐free endothelial growth medium (SFEGM) containing EBM‐2 (Lonza, Basel, Switzerland, http://www.lonza.com), 15% KnockOut Serum Replacement (KOSR; Thermo Fisher), 5 ng/ml FGF‐2, 10 ng/ml VEGF, 5 ng/ml EGF, 10 ng/ml IGF‐1, 1 U/ml heparin, 1 μg/ml hydrocortisone, and 20 μg/ml l‐ascorbic acid. The adhesive endothelial cells were cultured for 6 days before passaging and subcultured every 4 days.

### Measurement of TGF‐β Concentration in Media

The endothelial cells were cultured without SB431542 or exogenous TGF‐β1 for 24 hours, and then the supernatant from culture was collected. The human TGF‐β1 concentrations were measured by the enzyme‐linked immunosorbent assay (ELISA) kit (R&D Systems, Minneapolis, MN, https://www.rndsystems.com) following the manufacturer's instructions. The end‐point absorbencies were determined on iMark Microplate Absorbance Reader (Bio‐Rad Laboratories, Hercules, CA, http://www.bio‐rad.com).

### LDL‐Uptake Assay

hPSC‐ECs were cultured in SFEGM containing 10 ng/ml DiI‐acetylated low‐density lipoprotein (DiI‐Ac‐LDL; Thermo Fisher) for 6 hours. After being washed twice with phosphate‐buffered saline (PBS), the cells were examined by a fluorescence microscope.

### Vascular‐Like Network Forming Assay

The assay was performed essentially as previously described 10, 31. Briefly, 24‐well plates were coated with 200 μl per well Matrigel matrix (BD Biosciences) at room temperature for more than 30 minutes. Human umbilical vein endothelial cells (HUVECs) or hESC‐ECs (5 × 10^4^ cells) were plated on Matrigel‐coated plates in 500 μl EGM‐2 medium (Lonza) and incubated at 37°C in 5% CO_2_. The structures were photographed by a phase‐contrast microscopy after 16 hours of incubation. To quantify the capacity of angiogenesis, the total branch numbers of network structure in each well were manually counted under microscope as previously described 32. The quantification of branches was performed by using the software ImageJ, which is a Java‐based image‐processing program developed at the National Institutes of Health. Briefly, the branch length was measured as a “straight line” and calculated by pixels. Then the pixels were converted to micrometers according to the original photo magnification (×40) and resolution (pixels per inch).

### Immunocytochemistry

The cells were fixed with 4% paraformaldehyde in PBS at room temperature for 10 minutes, permeabilized with 0.1% Triton X‐100 in PBS at room temperature for 10 minutes, and then incubated with 1% BSA in PBS for 30 minutes to block nonspecific binding. The treated cells were then incubated with the following antibodies: CD144‐FITC and CD31‐PE for 1 hour at room temperature. The nuclei were stained by 0.1 μg/ml 4′,6‐diamidino‐2‐phenylindole (DAPI) for 3 minutes. The results were examined by a fluorescence microscope.

### Real‐Time Polymerase Chain Reaction Analysis

Total RNA from undifferentiated hPSCs and EBs at different time points were isolated by using Direct‐zol RNA MiniPrep kit (Zymo Research, Irvine, CA, http://www.zymoresearch.com). To eliminate DNA contamination, the RNA samples were treated with DNase I (RNase free) on the column, following manufacturer's instruction. Total RNA (1 μg) was used for each reverse transcription reaction with SuperScript III (Thermo Fisher). Real‐time quantitative polymerase chain reaction (qPCR) was performed on an iQ5 thermal cycler (Bio‐Rad), and glyceraldehyde‐3‐phosphate dehydrogenase was used as an internal standard for normalization.

### Flow Cytometric Analysis

Adherent cells or EBs were dissociated to form a single‐cell suspension by TrypLE (Thermo Fisher) treatment and washed with FACS buffer (1% fetal bovine serum and 1 mM EDTA in PBS). The dissociated cells were resuspended in FACS buffer and labeled with fluorochrome‐conjugated antihuman CD34‐APC, CD31‐PE, and CD144‐FITC antibodies. Isotype‐matched control antibodies were used to determine the background staining. Flow cytometry was performed on an LSR II analyzer (BD Biosciences). Data analysis was performed using FlowJo software or FCS Express software.

### Hind‐Limb Ischemic Mouse Model and Magnetic Resonance Imaging Analysis

NOD.Cg‐*Prkdc^scid^ Il2rg^tm1Wjl^*/SzJ (NSG) mice were obtained from Jackson Laboratory (Bar Harbor, ME, https://www.jax.org). The animal experiments were approved by the Institutional Animal Care and Use Committee at Maine Medical Center and Johns Hopkins University. The care of all experimental animals was in accordance with institutional guidelines. The surgery of hind‐limb ischemia was performed with minor modification of previous description 33. Briefly, 8‐ to 10‐week‐old NSG mice were anesthetized and put in the supine position over a heated pad on the operating table. An incision was made approximately 1 cm long starting from the knee to the medial thigh using fine forceps and surgical scissors. The femoral artery was carefully separated from the femoral vein and nerves at the proximal location near the groin, and then a strand of 7‐0 silk suture was passed underneath the proximal end of the femoral artery. Similarly, the femoral artery was also separated at the distal location close to the knee, and then a suture was passed underneath the distal end of the femoral artery proximal to the popliteal artery. The femoral artery was tightly occluded at both the proximal and distal ends using double knots. Cells (5 × 10^5^ in 100‐μl PBS) were injected into the muscle aside the intervening segment of ligated vessel, and then the incision was closed using 5‐0 vicryl sutures. The mice were closely monitored and maintained for 2 weeks.

The ischemia induction was checked by magnetic resonance imaging (MRI) at the indicated date. The MRI images were analyzed by Fiji software. For quantification of revascularization, Volume Calculator (VolCal), a plug‐in of Fiji software that is based on both vascular length and blood flow strength, was used.

### Cell Proliferation Assay

The proliferation of hPSC‐ECs was examined using a WST‐1 cell proliferation kit (Millipore, Billerica, MA, http://www.emdmillipore.com) following the manufacturer's instructions. Briefly, the endothelial cells at passage 3 were cultured in a 96‐well plate (2,000 cells per well) with indicated small molecular chemicals. After 2 days culture, the medium was changed to fresh SFEGM with WST‐1 reagent and incubated for 2 hours. The end‐point absorbencies were determined on iMark Microplate Absorbance Reader (Bio‐Rad). A standard curve was established by using endothelial cells at multiple cell densities.

### Cellular Senescence Assay

The detection of senescence‐associated β‐galactosidase (SA‐β‐gal) activity was used to examine cellular senescence. The SA‐β‐gal staining was performed by a cellular senescence assay kit (Millipore) following the manufacture's instruction. Briefly, the endothelial cells were treated with fixing buffer at room temperature for 10 minutes and then thoroughly washed with PBS. The cells were incubated with SA‐β‐gal detection solution at pH 6.0, 37°C without CO_2_, and protected from light overnight. The blue stained cells were checked under a phase‐contrast microscopy.

### Statistical Analysis

Experiments were repeated three times. Results were presented as means ± SD. The data were subjected to statistical analysis by unpaired Student's two‐tailed *t* test. Results with a value of *p* < .05 were considered statistically significant.

## Results

### Endothelial Cells Derived From hPSCs Had a Limited Expansion Capability

**Figure 1:**
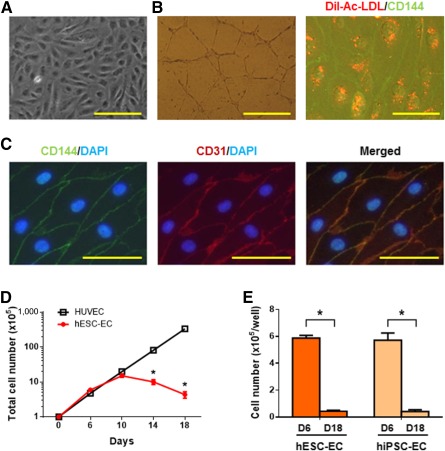
hPSC‐ECs had a limited expansion capability. The CD34^+^CD31^+^CD144^+^ cells from hESCs and hiPSCs were isolated after 8 to 10 days of embryoid body differentiation, and then were cultured in SFEGM to generate hPSC‐ECs. **(A):** Morphology of cobble‐like hPSC‐ECs. Scale bar = 200 μm. **(B):** Endothelial functional assays in vitro. Left: Vascular‐like network forming assay. 5 × 10^4^ ECs were plated in wells containing Matrigel with 0.5 ml SFEGM and incubated at 37°C in 5% CO_2_ for 16 hours. Scale bar = 500 μm. Right: Low‐density lipoprotein uptake assay. ECs were incubated with 10 μg/ml DiI‐AC‐LDL (red) for 6 hours and stained by anti‐CD144‐FITC (green) for fluorescent photograph. Scale bar = 50 μm. **(C):** Immunocytochemistry assay of hPSC‐ECs. ECs were stained by anti‐CD144‐FITC and anti‐CD31‐PE. The cell nuclei were detected by DAPI staining. Scale bar = 50 μm. **(D):** Proliferation of hESC‐ECs. The CD34^+^CD31^+^CD144^+^ cells (1 × 10^5^) from hESC‐ECs were cultured in SFEGM on collagen I‐coated wells. The number of cells was counted at different time points (red line). The HUVECs were used as a cell control (black line). The accumulated total cell numbers were calculated from each passage. **(E):** EC numbers at D6 and D18. The hESC‐ECs and hiPSC‐ECs were expanded as mentioned previously. At each passage, 1 × 10^5^ cells were cultured in one well of a 6‐well plate, and cell numbers were counted accordingly.∗, *p* < .05. Abbreviations: D, day; DAPI, 4′,6‐diamidino‐2‐phenylindole; DiI‐Ac‐LDL, DiI‐acetylated low‐density lipoprotein; EC, endothelial cell; hESC, human embryonic stem cell; hiPSC, human induced pluripotent stem cell; HUVEC, human umbilical vein endothelial cell; SFEGM, serum‐free endothelial growth medium.

We previously showed that CD34^+^ cells derived from hESCs are capable of generating functional endothelial cells 10, 11. Further study demonstrated that CD34^+^CD31^+^CD144^+^ cells derived from hESCs and hiPSCs contain HEPs that give rise to hematopoietic cells and endothelial cells 28, 34. Cultured in a serum‐free endothelial cell grow medium 28, the endothelial cells derived from hPSC‐derived HEPs had a cobblestone morphology (Fig. 1A) and expression of CD144 (VE‐cadherin) and CD31 (PECAM‐1) at the cell‐cell junction (Fig. 1C) that is crucial for the maintenance of vascular integrity 35, 36. Vascular‐like network formation assay and DiI‐acetylated low‐density lipoprotein uptake assays, which are used to characterize functional endothelial cells 37, were performed to confirm endothelial characteristics (Fig. 1B; supplemental online Fig. 2). Endothelial cells derived from two hESC lines (H1 and H9) and two hiPSC lines (HDFa‐YK26 and BC1) had similar endothelial features as HUVECs (supplemental online Fig. 1). The growth curve of hPSC‐ECs was different from that of HUVECs in a serum‐free endothelial cell growth medium. After three passages (day 14), hESC‐ECs stopped growing, whereas HUVECs maintained proliferation potential (Fig. 1D). Similar growth arrest was observed in hiPSC‐ECs, suggesting that hPSC‐ECs have limited capacity of cell expansion (Fig. 1E).

### Endothelial Cells After Prolonged Culture Decreased Endothelial Function

**Figure 2:**
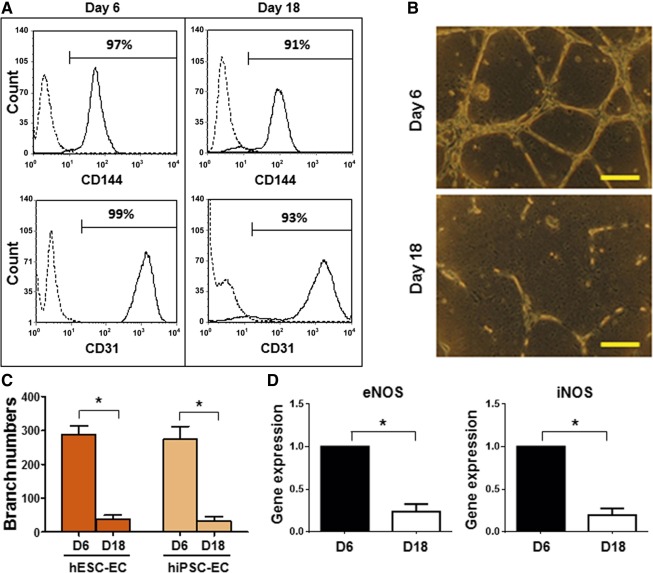
The endothelial function of hPSC‐ECs was attenuated after prolonged culture. The hPSC‐ECs were expanded as mentioned previously and were characterized at D6 and D18, respectively. **(A):** Fluorescence‐activated cell sorting analysis of CD144 and CD31 expression in hESC‐ECs. **(B):** Vascular‐like network forming assay of in vitro endothelial function. 5 × 10^4^ hESC‐ECs at D6 or D18 were plated in wells containing Matrigel with 0.5 ml serum‐free endothelial growth medium and incubated at 37°C in 5% CO_2_ for 16 hours. Scale bar = 200 μm. **(C):** The branch numbers were counted after 16 hours of Matrigel assay. **(D):** Quantitative polymerase chain reaction assay of gene expression of eNOS and iNOS in hPSC‐ECs at day 6 or day 18. The relative gene expression was normalized by expression of Glyceraldehyde 3‐phosphate dehydrogenase. ∗, *p* < .05. Abbreviations: D, day; EC, endothelial cell; eNOS, endothelial nitric oxide synthase; hESC, human embryonic stem cell; hiPSC, human induced pluripotent stem cell; iNOS, induced nitric oxide synthase.

To test whether endothelial cells maintain their function after prolonged culture, we examined the expression of CD144 and CD31 in endothelial cell culture on day 6 (passage 1 [P1]) and day 18 (passage 4 [P4]) by flow cytometry. On day 6, almost all endothelial cells expressed both CD144 and CD31. By day 18, the hPSC‐ECs maintained high expressions of CD144 and CD31 (91% and 93% respectively) (Fig. 2A), suggesting that most of the cells remained an endothelial feature. To characterize the functionality of endothelial cells after prolonged culture, we examined whether hPSC‐ECs were capable of vascular‐like network formation in vitro. The hPSC‐ECs from day 6 (P1) and day 18 (P4) were loaded onto Matrigel, and the vascular network branches were quantified after 16 hours, as we previously described 11. As shown in Figure 2B, endothelial cells from day 18 lost their ability to assemble into vascular network. The cord formation of endothelial cells from day 18 was significantly reduced and appeared as short cords (Fig. 2C; supplemental online Fig. 3A). Endothelial cell proliferation and migration are critical events for angiogenesis, in which endothelium‐derived nitric oxide (eNO) plays an essential role 38. To determine whether prolonged culture of hPSC‐ECs affects eNO production, we analyzed eNO synthase (eNOS) expression by qPCR. As shown in Figure 2D, the expression of eNOS and induced nitric oxide synthase (iNOS) in hPSC‐ECs from day 18 was significantly decreased, compared with those from day 6. We harvested culture media and measured the concentration of nitrate (NO_3_^‐^) and nitrite (NO_2_^‐^), which are breakdown products of nitric oxide (NO), by NO assay. The concentration of nitrate and nitrite was normalized by cell numbers (supplemental online Fig. 4A). Our data demonstrated that the effect of aging hPSC‐ECs on NO production was correlated with nitric oxide synthase (NOS) expression. Taken together, these data indicated that hPSC‐ECs lost functionality after prolonged culture.

### Cellular Senescence Increased in hPSC‐ECs After Prolonged Culture

**Figure 3:**
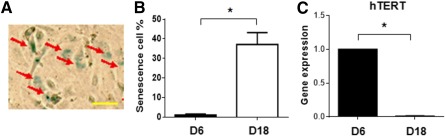
hPSC‐ECs underwent cellular senescence after prolonged culture. **(A):** SA‐β‐gal staining on hESC‐ECs at day 18. Senescent cells were positive for SA‐β‐gal staining (red arrows). Scale bar = 100 μm. **(B):** Statistic percentages of senescent hPSC‐ECs on day 6 and day 18. **(C):** Gene expression of a telomerase activity‐related gene hTERT by quantitative polymerase chain reaction assay. The relative gene expression was normalized by expression level on day 6. ∗, *p* < .05. Abbreviations: D, day; hTERT, human telomerase reverse; hPSC, human pluripotent stem cell; EC, endothelial cell; SA‐β‐gal, senescence‐associated β‐galactosidase.

NO, synthesized by NOS, in endothelial cells prevents endothelial cellular senescence 39. Because hPSC‐ECs from day 18 have a decreased NOS expression as well as attenuated functionality, we hypothesized that hPSC‐ECs after prolonged culture obtain cellular senescence. SA‐β‐gal activity at pH 6.0 is widely accepted as a biomarker of cellular senescence 40, 41, 42. We performed a cytochemical assay of SA‐β‐gal to evaluate the status of senescence in endothelial cells. SA‐β‐gal positive cells were observed in hPSC‐ECs for 18 days (Fig. 3A). Compared with hPSC‐ECs from day 6 at which SA‐β‐gal positive cells were occasionally detected, the proportion of SA‐β‐gal positive cells from day 18 hPSC‐ECs increased dramatically (Fig. 3B; supplemental online Fig. 5A). Notably, the hPSC‐ECs at day 18 had a morphology of an increased cell size, compared with those at day 6 (supplemental online Fig. 5A). Taken together, the data indicated a significant increase of senescent endothelial cells at day 18.

Shortening telomere length has been demonstrated to trigger the onset of senescence, and cellular senescence usually accompanies a decreased telomerase activity that can synthesize new telomeric repeats and restore telomere length 39, 43, 44. We harvested hPSC‐ECs on day 6 and day 18 of culturing, and performed qPCR analysis. The expression of hTERT, a catalytic subunit of the telomerase, was significantly decreased in hPSC‐ECs after prolonged culture (day 18), compared with hPSC‐ECs from day 6 (Fig. 3C). These data indicated that an increased senescence is associated with a decreased expression of hTERT in endothelial cells.

### Inhibition of TGF‐β Signaling Reduced Endothelial Cellular Senescence and Promoted Cell Growth

**Figure 4:**
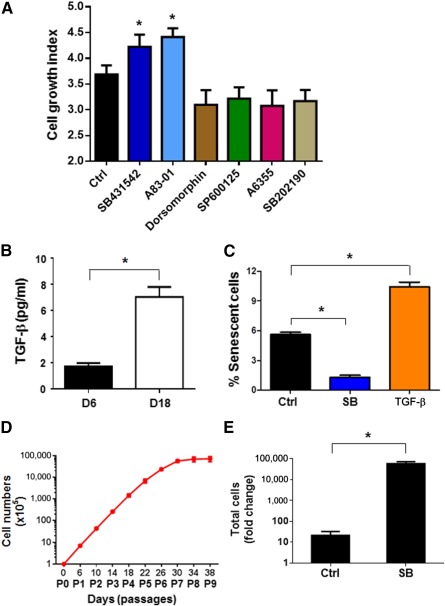
Inhibition of TGF‐β signaling promoted hPSC‐EC proliferation. **(A):** Cell proliferation assay. The hPSC‐ECs on D6 were cultured with a variety of inhibitors for 2 days to attain logarithmic growth phase, and then the cell proliferation was measured via WST‐1 assay. **(B):** Quantification of endogenous TGF‐β1 expression. The hPSC‐ECs on day 6 and day 18 were cultured in fresh SFEGM for 24 hours, and then the concentration of TGF‐β1 in supernatant medium was measured by enzyme‐linked immunosorbent assay. The quantification of TGF‐β1 was normalized per 10^4^ cells. **(C):** Cellular senescence analysis. The hPSC‐ECs D6 were cultured with 10 µM SB431542 or 2 ng/ml TGF‐β1 for 4 days, and then the senescent cells were detected by senescence‐associated β‐galactosidase staining. **(D):** Growth curve of hESC‐EC expansion. The hPSC‐ECs were cultured in SFEGM with 10 µM SB431542, and the accumulated total cell numbers during expansion were calculated from each passage. **(E):** Fold increase of total cell number. The hPSC‐ECs were cultured with or without SB431542 to reach the maximal proliferation respectively, and the fold change of cell expansion was calculated accordingly. ∗, *p* < .05 compared with the control. Abbreviations: Ctrl, control; D, day; EC, endothelial cell; hPSC, human pluripotent stem cell; P, passage; SB, SB431542; SFEGM serum‐free endothelial growth medium; TGF‐β, transforming growth factor β.

To identify signaling pathways that regulate endothelial senescence, we screened small molecular inhibitors for the ones that promote hPSC‐EC proliferation by WST‐1 assay 45. The hPSC‐ECs (P2) were cultured for 48 hours in the presence of inhibitors, and the cell growth indexes were measured by incubating the cells for 2 hours in the presence of WST‐1 reagents. As shown in Figure 4A, SB431542 and A83‐01, which specifically blocks the activity of TGF‐β type I receptors, but not BMP type I receptors 46, significantly increased the growth index of hPSC‐ECs. An addition of dorsomorphin (BMP4 signaling inhibitor), SP600125 (JNK inhibitor), SB202190 (p38 MAPK inhibitor), or A655 (ERK inhibitor) reduced the growth index modestly (Fig. 4A).

**Figure 5:**
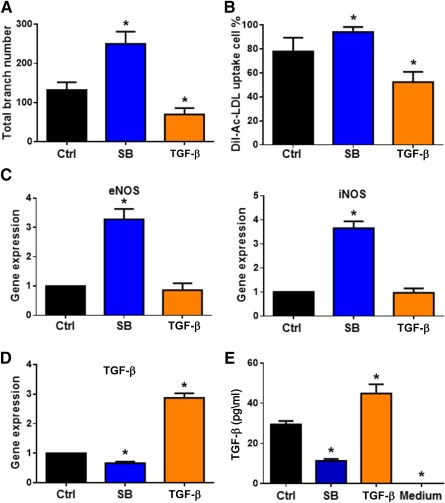
Inhibition of TGF‐β signaling preserved hPSC‐EC function in vitro. The hPSC‐ECs were cultured in SFEGM with SB431542 for 18 days and then subcultured with 10 µM SB431542 or 2 ng/ml TGF‐β1. The SFEGM medium without additions of SB431542 and TGF‐β1 was used as control. After 4 days of culture, the cells were harvested for in vitro function and gene expression assays. **(A):** Vascular‐like network forming assay quantified by network branch number. **(B):** Low‐density lipoprotein uptake assay quantified by percentage of DiI‐Ac‐LDL‐positive cells. **(C):** Relative gene expressions of eNOS and iNOS by qPCR assay. **(D):** The 1 × 10^5^ hPSC‐ECs were subcultured in 2 ml of SFEGM, and with SB431542 or TGF‐β1 respectively, for 2 days, and then the medium was changed to fresh SFEGM. After 24 hours culture, the cells were harvested for detection of TGF‐β gene expression via qPCR. **(E):** The supernatant medium was collected to measure secreted TGF‐β1 by enzyme‐linked immunosorbent assay. ∗, *p* < .05, compared with the control. Abbreviations: Ctrl, control; DiI‐Ac‐LDL, DiI‐acetylated low‐density lipoprotein; EC, endothelial cell; eNOS, endothelial nitric oxide synthase; hPSC, human pluripotent stem cell; iNOS, induced nitric oxide synthase; qPCR, quantitative polymerase chain reaction; SB, SB431542; SFEGM, serum‐free endothelial growth medium; TGF‐β, transforming growth factor‐β.

It has been reported that TGF‐β signaling negatively regulates the expression of hTERT 47, 48 and plays an important role in promoting senescence in several cell types 49, 50, 51, 52, 53, 54, 55. To examine TGF‐β expression during hPSC‐EC culture, we measured TGF‐β1 protein secreted into culture by ELISA. We observed that the secretion of TGF‐β1 protein was significantly higher in hPSC‐ECs from day 18 than those from day 6 (Fig. 4B), whereas TGF‐β protein was undetectable in SFEGM (Fig. 5E). To test whether inhibition of TGF‐β signaling reduces the cellular senescence of hPSC‐ECs, we cultured hPSC‐ECs in the presence of TGF‐β1 or SB431542 (TGF‐β inhibitor) for 4 days and stained the senescent cells with SA‐β‐gal at pH 6.0. The number of senescent cells increased in the presence of TGF‐β1 and decreased in the presence of SB431542 (Fig. 4C; supplemental online Fig. 5B), indicating that suppression of TGF‐β signaling prevents hPSC‐ECs from undergoing senescence. In the presence of SB431542, hPSC‐ECs continued expansion until day 30 (passage 7) in the serum‐free endothelial medium (Fig. 4D). Compared with an approximate 20‐fold expansion of hPSC‐ECs without SB431542, suppression of TGF‐β signaling by SB431542 during hPSC‐EC culture resulted in a 5.6 × 10^4^ folds expansion of the endothelial cells (Fig. 4E).

### Endothelial Function Was Preserved by Inhibition of TGF‐β Signaling

To examine the functional relationship between TGF‐β signaling and endothelial function of hPSC‐ECs, we subcultured the hPSC‐ECs with SB431542 or TGF‐β1 for 4 days. The hPSC‐ECs were subjected to a vascular network forming assay on Matrigel. The vascular network formatting capability of hPSC‐ECs was significantly increased in the presence of SB431542 (Fig. 5A), whereas endothelial cells cultured with TGF‐β1 decreased their ability to assemble into a vascular network and appeared in clumps of round cells or short cords (supplemental online Figs. 3B, 6A). To characterize functional endothelial cells, we also performed DiI‐Ac‐LDL uptake assays (supplemental online Fig. 6B) as we previously described 27, 28. The endothelial cells grown in the presence of SB431542 increased their ability to uptake DiI‐Ac‐LDL (Fig. 5B). Furthermore, the gene expressions of eNOS and iNOS, as well as the total NO products (nitrate and nitrite), increased in hPSC‐ECs in the presence of SB431542 (Fig. 5C; supplemental online Fig. 4B). In addition, SB431542 abated endogenous TGF‐β1 expression, whereas exogenous TGF‐β1 produced a reverse effect (Fig. 5D, 5E), suggesting TGF‐β signaling plays a positive feedback role in loss of functionality of hPSC‐ECs.

**Figure 6:**
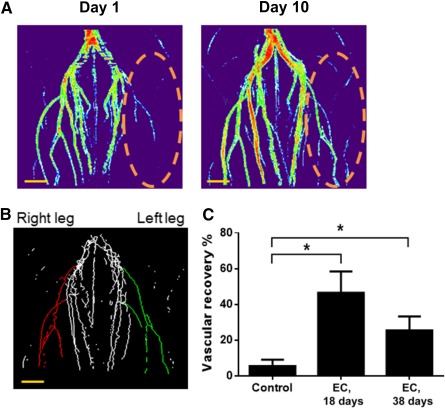
hPSC‐ECs promoted vascular recovery in hind‐limb ischemic mice. **(A):** Thermal images of blood vessel structure on hind‐limb ischemic mice. The hind‐limb ischemia was induced by vascular ligation of left femoral artery via surgery on NOD.Cg‐*Prkdc^scid^ Il2rg^tm1Wjl^*/SzJ mice. The hPSC‐ECs were expanded in serum‐free endothelial growth medium with SB431542 for 18 days and transplanted into the ischemic leg (5 × 10^5^ cells per mouse). The revascularization in ischemic tissues was detected by MRI technology on day 1 and day 10 after endothelial cell transplantation. The MRI data were analyzed by Fiji software. **(B):** Quantification of revascularization in ischemic tissue on day 10 after endothelial cell transplantation. The MRI data were analyzed by Fiji software using a plug‐in of VolCal to quantify vascular length and blood flow strength on ischemic leg (left, green) and control leg (right, red). **(C):** Statistic quantification of vascular recovery 10 days after endothelial cell transplantation. The vascular‐promoting function of hPSC‐ECs expanded with SB431542 on day 18 and day 38 were assessed, respectively. The hind‐limb ischemic mice without endothelial cell transplantation were used as a control. Scale bar = 2 mm. ∗, *p* < .05. Abbreviations: EC, endothelial cell; hPSC, human pluripotent stem cell; MRI, magnetic resonance imaging.

We previously demonstrated that hESC‐ECs are capable of self‐assembling into functional blood vessels that spontaneously integrate into the circulatory system of a mouse model 10. To test whether endothelial cells cultured in the presence of SB431542 are capable to promote revascularization in vivo, we assessed the vascular regenerative ability by implanting hPSC‐ECs into hind‐limb ischemic mice. The left femoral artery of NSG mice was ligated to induce ischemia. The hPSC‐ECs cultured in the presence of SB431542 for 18 days or 38 days were injected into the gastrocnemius muscle of ischemic legs. The revascularization in ischemic tissues was monitored and quantified by magnetic resonance imaging (MRI) technology (Fig. 6A). The blood vessels were quantified and compared in left and right legs for the same mice after 10 days of implantation (Fig. 6B). As shown in Figure 6C, the hPSC‐ECs after prolonged culture with SB431542 until day 38, maintained the ability to enhance vascular recovery in vivo, albeit early passages of hPSC‐ECs were more efficient to revascularize ischemia tissues.

### TGF‐β Signaling Regulated Cell Cycle of Senescent hPSC‐ECs

**Figure 7:**
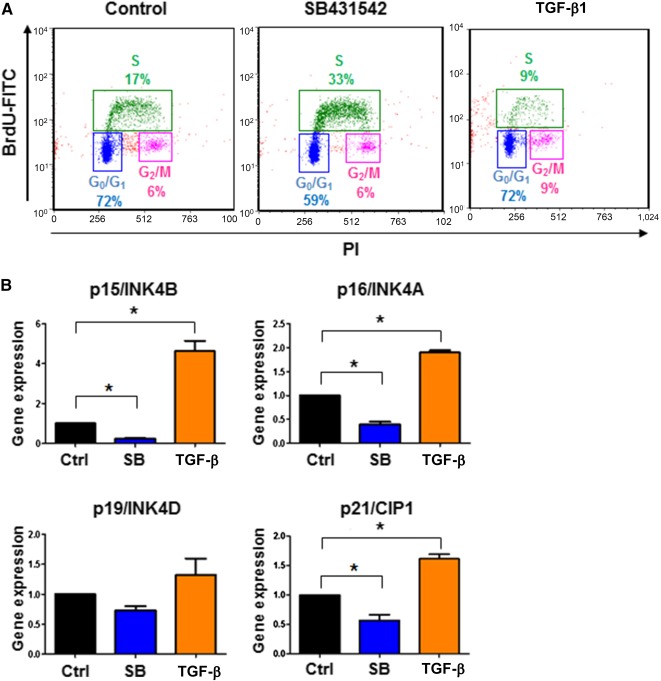
TGF‐β signaling regulated cell cycle of senescent hPSC‐ECs. The hPSC‐ECs were cultured in SFEGM with SB431542 for 18 days and then subcultured in SFEGM with 10 µM SB431542 or 2 ng/ml transforming growth factor‐β1, respectively, for 2 days. The cells were harvested for cell cycle and gene expression analyses. **(A):** Cell cycle analysis by FACS. The hPSC‐ECs were pulse‐labeled with 20 µM BrdU at 37°C for 30 minutes and then further incubated with anti‐BrdU‐FITC and PI for FACS analysis. **(B):** Gene expressions analysis of cyclin‐dependent kinase inhibitors. The hPSC‐ECs were harvested to make RNA for gene expression analysis by quantitative polymerase chain reaction. ∗, *p* < .05. Abbreviations: BrdU, bromodeoxyuridine; EC, endothelial cell; hPSC, human pluripotent stem cell; FACS, fluorescence‐activated cell sorting; FITC, fluorescein isothiocyanate; PI, propidium iodide; SFEGM, serum‐free endothelial growth medium; TGF‐β, transforming growth factor‐β.

Irreversible growth arrest in senescent cells occurs in the transition from G_1_ phase to S phase of the cell cycle 56. To investigate how TGF‐β inhibition affects the progressive loss of endothelial proliferative capacity (replicative senescence) during culturing, we examined the cell cycle of hPSC‐ECs. The hPSC‐ECs were cultured in the presence of SB431542 for 18 days (P4) and then subcultured for 48 hours with SB431542 or TGF‐β1. The cells were then pulse‐labeled with bromodeoxyuridine (BrdU) for 30 minutes. To quantify the proportion of cells in each phase of the cell cycle, flow cytometry was used after propidium iodide staining. As shown in Figure 7A, inhibition of TGF‐β signaling by SB431542 resulted in more cells in S phase and less cells in G_1_ phase, compared with the control. Cell cycle is regulated by an array of cyclin‐dependent kinase inhibitors (CDKIs). To examine the effect of TGF‐β inhibition on the expression of CDKIs in hPSC‐ECs, we subcultured hPSC‐ECs (P4) for 48 hours with SB431542 or TGF‐β1 and tested CDKI expression levels by real‐time qPCR analysis (Fig. 7B). The gene expression of p15INK4B, p16INK4A, and p21CIP1 were substantially downregulated by SB431542 and upregulated by TGF‐β1, whereas the gene expression of p19/INK4D was moderately downregulated by SB431542 and upregulated by TGF‐β1. Taken together, these data suggested that the inhibition of TGF‐β signaling enhanced the S phase of the cell cycle via regulating CDKIs and promoted the proliferative capacity of hPSC‐ECs.

## Discussion

Vascular aging is a major risk factor for the development of cardiovascular diseases. Endothelial senescence is a cellular process that is clearly linked to both aging and the development of vascular pathologies 57, 58, 59. Senescent cells accumulate in normal arterial tissue over the life span of humans 60, 61 and in vascular damaged tissues 62, 63. It has been reported that the clearance of senescent cells reversed aged and diseased phenotypes in mouse models 64. Understanding human vascular aging will provide insights into the prevention of vascular diseases in older adults.

Development of therapeutic potential in hPSC‐ECs is hindered by the limited expansion potential of hPSC‐ECs. Previously, we generated hPSC‐ECs 27, 28, 65 and demonstrated that these ECs formed functional blood vessels in immunodeficient mice by cell transplantation 10. In the present study, we delineated the proliferative properties of hPSC‐ECs and used hPSC‐ECs as a model to investigate vascular cellular aging (senescence) in vitro. In contrast to human primary ECs such as HUVECs, the hPSC‐ECs quickly lost their proliferative potential, which was accompanied by a morphological change with a larger cellular size, as well as by attenuated cellular functions (Figs. 1, 2).

NO, produced by the enzyme eNOS, is one of the most important vasodilator and antiatherosclerotic molecules produced by the endothelium 66. Aged ECs have a reduced rate of NO synthesis, rendering them more sensitive to apoptotic stimuli 67. Expression of iNOS in endothelial cells is critical for protecting against oxidative damage 68. Our study demonstrated that aged hPSC‐ECs had a decreased expression of eNOS and iNOS, resulting in a decreased production of NO. Although the expression of EC markers, CD31 and CD144, remained high in prolonged cultured hPSC‐ECs, their vascular functions, such as network formation in Matrigel and NO production, were significantly decreased, correlated with an increased cellular aging. During cellular aging (senescence), the activity of lysosomal hydrolase β‐galactosidase is specifically increased in senescent cells at pH 6.0 41, 69, whereas the expression of telomerase is decreased, resulting in shortening of telomeres, the physical ends of chromosomes 70, 71, 72. The observations of downregulated eNOS/iNOS and telomerase reverse transcriptase, along with upregulated senescence‐associated β‐galactosidase (Figs. 2, 3), supported our hypothesis of senescent hPSC‐ECs after prolonged culture.

It has been shown that TGF‐β1 induces senescence and reactive oxygen species production in human primary cells, such as bone marrow mesenchymal stem cells 51, human mammary epithelial cells 53, and in tumor cells, such as in hepatocellular carcinoma 49 and myeloid tumor cells 52. TGF‐β signaling pathway is involved in angiogenesis and vascular disease 73. SB431542 is a specific inhibitor, working as a competitive adenosine 5′‐triphosphate binding site to inhibit the TGF‐β type I receptors, ALK4, ALK5, and ALK7, and has no effect on BMP receptors, ALK 2, ALK3, and ALK6 74. Our study demonstrated that inhibition of autocrine TGF‐β signaling by SB431542 reduced the number of senescent ECs, increased growth index, promoted enhanced vascular network formation, and enhanced eNOS/iNOS expression (Figs. 4, 5). Furthermore, the ability of revascularization in ischemic tissues of an animal model was enhanced when transplanted hPSC‐ECs were cultured in the presence of SB431542 (Fig. 6), although our study of experimental hind‐limb ischemic mice using MRI technology could not distinguish revascularization from endogenous or exogenous vascular cells. These data suggested that TGF‐β signaling facilitates senescence of hPSC‐ECs, which could be rescued by inhibition of TGF‐β signaling during cell culture in vitro.

Cellular senescence or cellular aging is permanent cell cycle arrest that primarily occurs in the G_0_/G_1_ 14. Our study of cell cycle analysis indicated that inhibition of TGF‐β released cells from G_0_/G_1_‐phase and increased cells in S phase. Furthermore, the expressions of CDKIs, p15/INK4B, p16/INK4A, and p21/CIP1, were suppressed by inhibition of TGF‐β signaling (Fig. 7), suggesting that TGF‐β signaling regulates hPSC‐EC senescence and proliferation through CDKIs intermediates.

A recent study showed that TGF‐β signaling promoted endothelial‐to‐mesenchymal transition, resulting in ECs losing their cell‐specific markers 75. We found that there was a small proportion of nonendothelial cells (CD31 and CD144 negative) after 18 days of culture (Fig. 2A). To examine whether non‐ECs after prolonged culture contribute to decreased vascular function, we purified the hPSC‐ECs by CD31 positive selection after 18 days of culture. As shown in supplemental online Figure 7, more than 99% of ECs after purification expressed EC markers, CD31 and CD144. The purified ECs after prolonged culture did not enhance endothelial phenotype and function.

## Conclusion

Our data elucidate that TGF‐β signaling negatively regulates endothelial cell function by inducing cellular senescence of hPSC‐ECs. Blockage of TGF‐β signaling suppressed the expression of p15INK4B, p16INK4A, and p21CIP1 and enhanced endothelial cell proliferation and function, including NOS expression, vascular network formation in vitro, and revascularization in ischemic tissue in mice.

## Author Contributions

H.B.: conception and design, collection and/or assembly of data; Y.G. and D.L.H.: collection and/or assembly of data; T.C.: conception and design, manuscript writing; Z.Z.W.: conception and design, data analysis and interpretation, manuscript writing, final approval of manuscript.

## Disclosure of Potential Conflicts of Interest

The authors indicated no potential conflicts of interest.

## Supporting information

Supporting Information
