# Supplementary material for: Suppression of Transforming Growth Factor‐β Signaling Delays Cellular Senescence and Preserves the Function of Endothelial Cells Derived from Human Pluripotent Stem Cells
Source: Stem Cells Transl Med. 2016 Sep 20;6(2):589–600. doi: 10.5966/sctm.2016-0089 (PMC5442820; doi:10.5966/sctm.2016-0089)
Supplement: Supplementary file 1 — Supporting Information [file SCT3-6-589-s001.pdf]

Supplemental Figures – Wang et al.

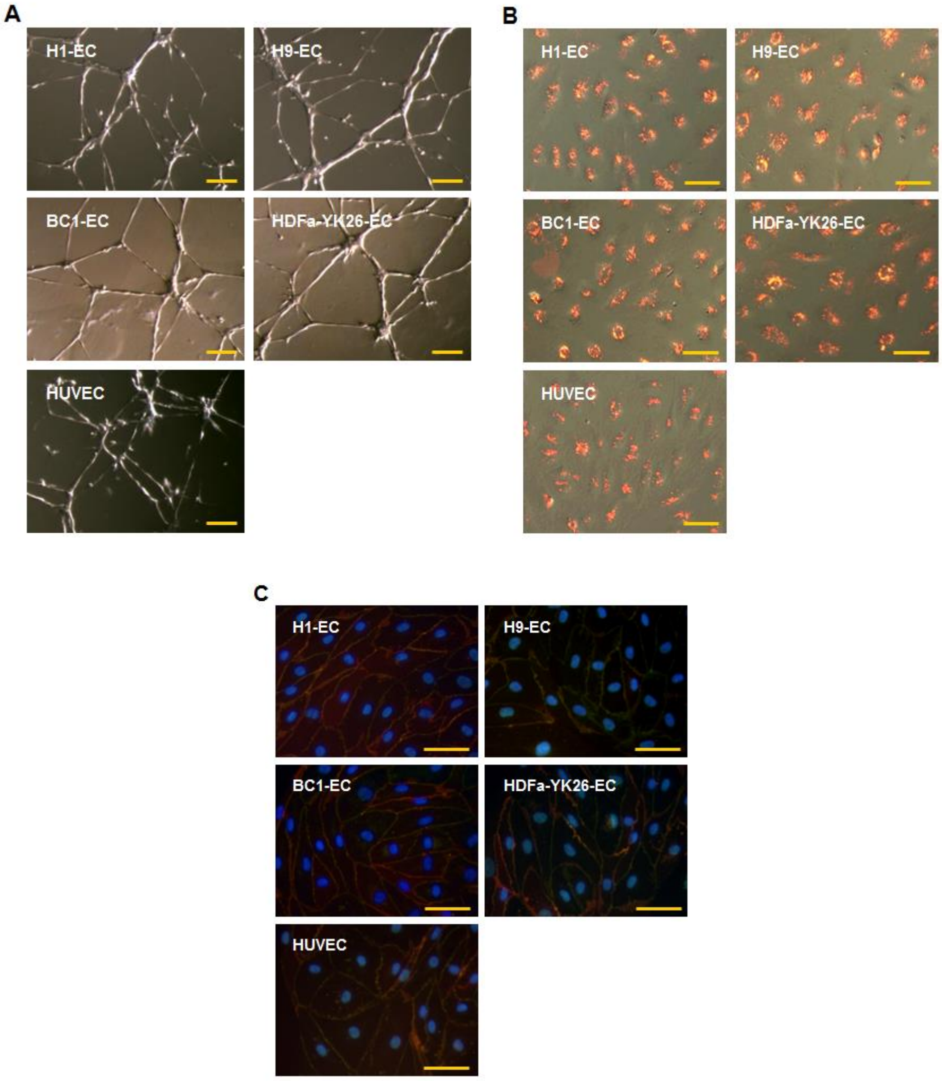

Fig. S1 Characterization of hPSC-ECs. A variety of hPSC-ECs were generated from $CD34^{+}CD31^{+}CD144^{+}$ cells of differentiated hESCs and hiPSCs. (A) Vascular-like network forming assay. $5x10^{4}$ ECs were plated in wells containing Matrigel with 0.5 ml SFEGM, and normally cultured for 16 hours. Photo size bar = 100 μm. (B) LDL-uptake assay. ECs were incubated with 10 ug/ml Dil-Ac-LDL (red) for 6 hours, and the images were shown as combination of light phase and fluorescence phase. Photo size bar = 50 μm. (C) Immunocytochemistry assay. ECs were stained by anti-CD144-FITC and anti-CD31-PE. The cell nuclei were detected by DAPI staining. The images were shown as combination of fluorescent photographs. Photo size bar = 50 μm.

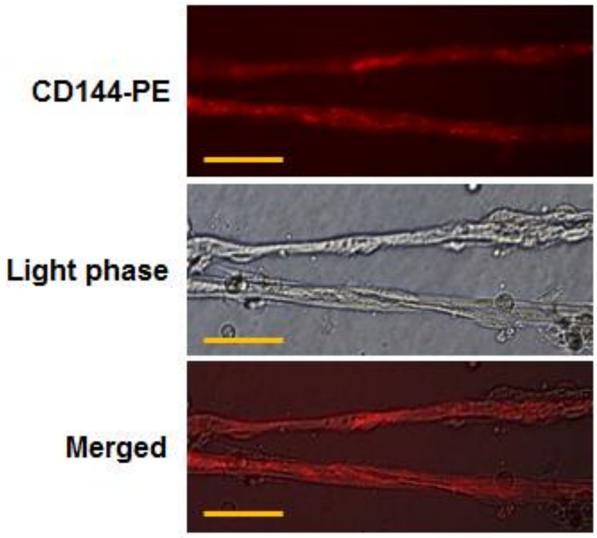

Fig. S2 Immunocytochemistry assay of vascular-like tubes. The hPSC-ECs were utilized to form vascular-like tubes on Matrigel. The cells in tubes were stained by anti-human CD144-PE (Miltenyi Biotech). A representative morphology was shown by combination of light phase and fluorescent phase photographs. Photo size bar = 50 μm.

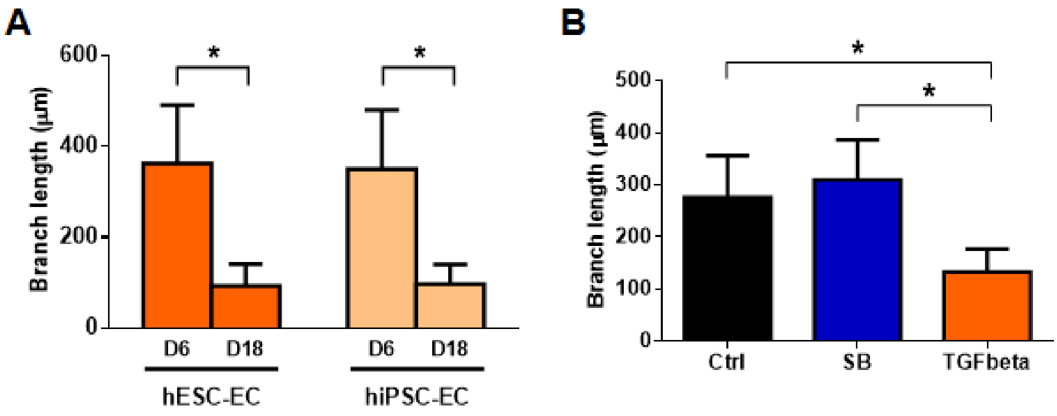

Fig. S3 Quantification of branch length of vascular-like tubes. The hPSC-ECs were utilized to form vascular-like tubes on Matrigel. To quantify the vascular-like tubes, the branch length was measured as 'Straight Line' and calculated as pixels by using ImageJ software. And then the pixels were converted to [illegible]m according to the original photo magnification (40 x) and resolution (pixels/inch). (A) The branch length of vascular-like tubes formed by hESC-ECs and hiPSC-ECs at day 6 and day 18, respectively. (B) The hPSC-ECs were cultured in SFEGM with SB431542 for 18 days, and then sub-cultured with 10 µM SB431542 or 2 ng/ml TGF-[illegible]1. Normal medium without SB431542 or TGF-[illegible]1 was used as a control. After 4 days of culture, the cells were harvested for vascular-like network forming assay in Matrigel. The branch length of vascular-like tubes was quantified as above-mentioned. * $p<0.05$.

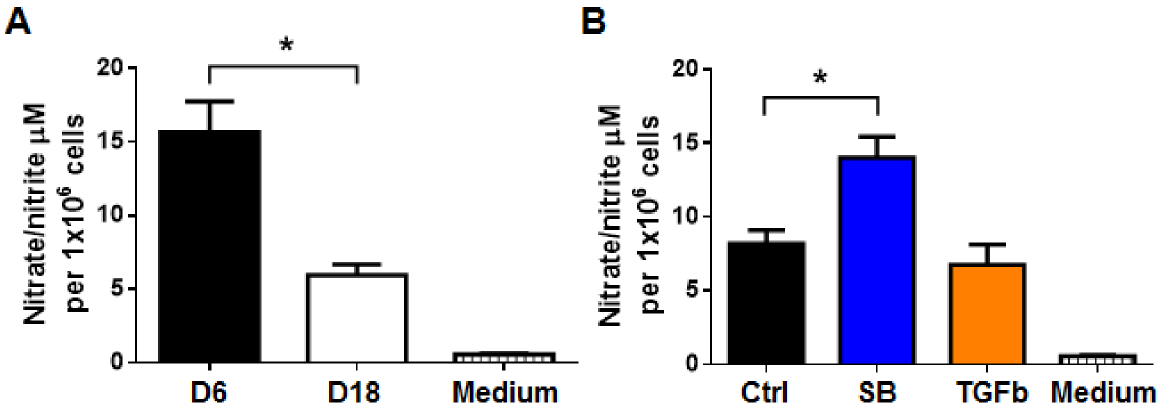

Fig. S4 Nitric oxide (NO) assay of hPSC-ECs. The endothelial medium supernates were collected after 24 hours culture with or without SB431542 and TGF-□. Meanwhile, the cell numbers of hPSC-ECs were counted. The total NO production was quantified as the sum of both nitrate and nitrite using a nitrate/nitrite colorimetric assay kit (Cayman Chemical). The results were presented as □M per million cells. (A) NO assays of hPSC-ECs at day 6 and day 18 respectively. (B) The hPSC-ECs were cultured in SFEGM with SB431542 for 18 days, and then sub-cultured with 10 μM SB431542 or 2 ng/ml TGF-□1. Normal medium cultured for hPSC-ECs without SB431542 or TGF-□1 was used as a control. The medium without culture was used as a reference. After 4 days of culture, the culture media were harvested for NO assays. * $p<0.05$.

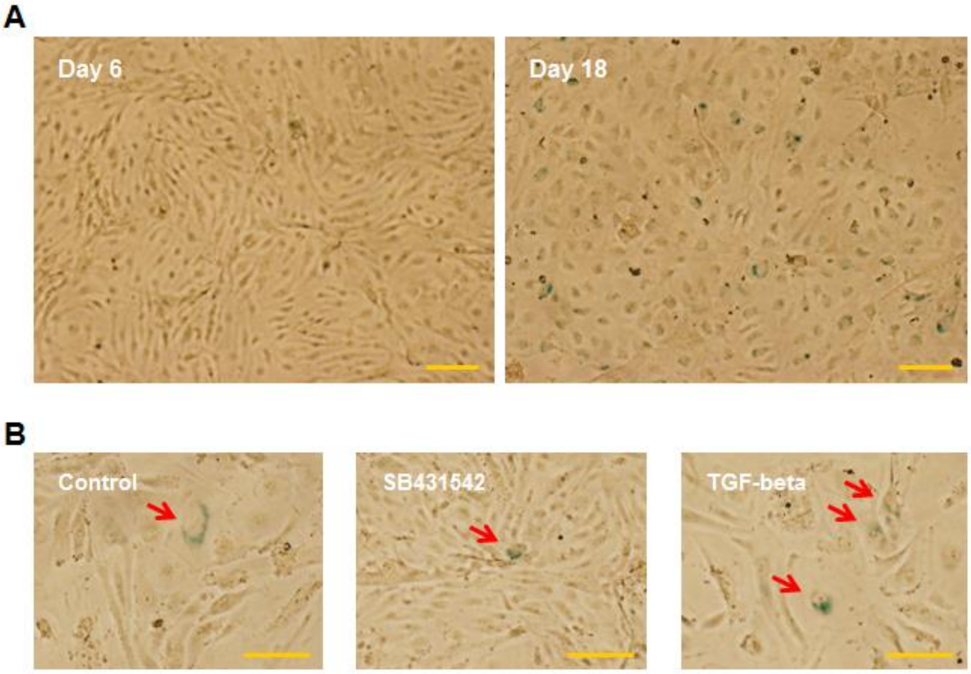

Fig. S5 Cellular senescence assay of hPSC-ECs. The senescence-associated β-galactosidase (SA-β-gal) staining was used to examine the senescent hPSC-ECs after either prolonged culture or regulating TGF-b signaling. (A) hPSC-ECs were stained at day 6 and day 18. (B) The hPSC-ECs were cultured in SFEGM with SB431542 for 18 days, and then sub-cultured with 10 μM SB431542 or 2 ng/ml TGF-1. EC culture in SFEGM without SB431542 or TGF-1 was used as a control. Photo size bar = 100 m.

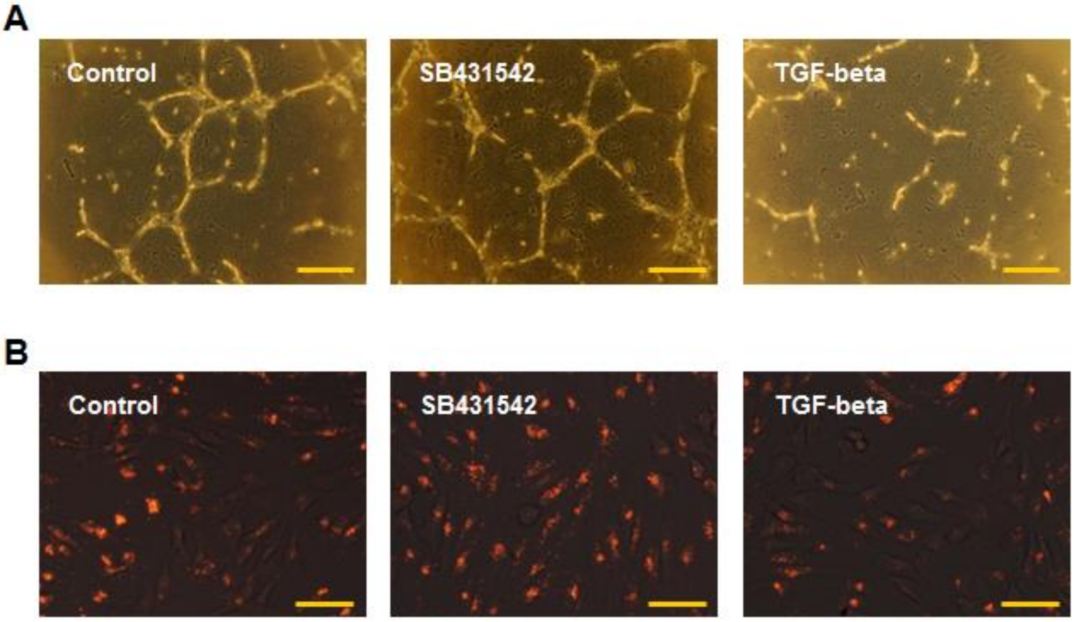

Fig. S6 Analyses of endothelial functionality of hPSC-ECs with and without TGF-β signaling manipulation. The hPSC-ECs were cultured in SFEGM with SB431542 for 18 days, and then sub-cultured with 10 μM SB431542 or 2 ng/ml TGF-β1. Normal medium without SB431542 or TGF-β1 was used as a control. After 4 days of culture, the cells were harvested for analyses. (A) Vascular-like network forming assay in Matrigel. Photo size bar = 200 μm. (B) LDL-uptake assay. The images were shown as combination of fluorescent phase (Dil-Ac-LDL, red) and light phase. Photo size bar = 50 μm.

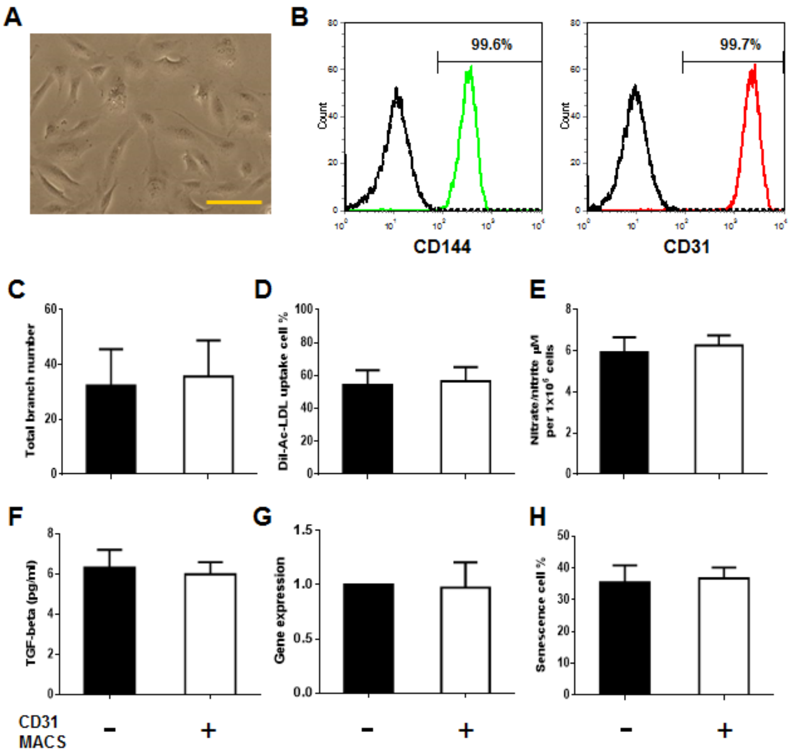

Fig. S7 Endothelial characterization of hPSC-ECs after purification. The hPSC-ECs had been cultured for 18 days, and the non-endothelial cells were excluded by MACS (magnetic-activated cell sorting) using anti-CD31 magnetic microbeads (Miltenyi Biotech). The endothelial properties of purified hPSC-ECs were characterized. (A) Morphology of hPSC-ECs after EC purification. Photo size bar = 50 μm. (B) Flow cytometric analyses of hPSC-ECs after EC purification. (C) Quantification of branch number of vascular-like network on Matrigel before (-) and after (+) EC purification. (D) LDL-uptake assay. (E) NO assay represented by total nitrate and nitrite. (F) TGF-β secretion measurement. (G) Gene expression of human TERT by QPCR analysis. (H) Quantification of cellular senescence by SA-β-gal staining.
